# Effects of antifungal stewardship using therapeutic drug monitoring in voriconazole therapy on the prevention and control of hepatotoxicity and visual symptoms: A multicentre study conducted in Japan

**DOI:** 10.1111/myc.13129

**Published:** 2020-06-25

**Authors:** Yukihiro Hamada, Takashi Ueda, Yoshitsugu Miyazaki, Kazuhiko Nakajima, Keiko Fukunaga, Taiga Miyazaki, Nana Nakada‐Motokawa, Miki Nagao, Hideki Kawamura, Akari Shigemi, Fumiya Ebihara, Toshimi Kimura, Kazuhiro Ikegame, Motoi Uchino, Hiroki Ikeuchi, Yoshio Takesue

**Affiliations:** ^1^ Department of Pharmacy Tokyo Women's Medical University Hospital Tokyo Japan; ^2^ Department of Infection Control and Prevention Hyogo College of Medicine Nishinomiya Japan; ^3^ Department of Chemotherapy and Mycoses National Institute of Infectious Diseases Tokyo Japan; ^4^ Department of Hematology Hyogo College of Medicine Nishinomiya Japan; ^5^ Department of Respiratory Medicine Nagasaki University Hospital Nagasaki Japan; ^6^ Department of Infection Control and Prevention Kyoto University Hospital Kyoto Japan; ^7^ Division of Medical and Environmental Safety Department of Infection Control and Prevention Kagoshima University Hospital Kagoshima Japan; ^8^ Department of Surgery Hyogo College of Medicine Nishinomiya Japan

**Keywords:** antifungal stewardship, hepatotoxicity, therapeutic drug monitoring, visual symptoms, voriconazole

## Abstract

**Background:**

Hepatotoxicity and visual symptoms are common adverse effects (AEs) of voriconazole therapy.

**Objective:**

To retrospectively evaluate the effects of treatment modification based on therapeutic drug monitoring on AEs in patients undergoing voriconazole therapy.

**Methods:**

The target voriconazole trough concentration (*C*_min_) was 1‐5 µg/mL. Receiver operating characteristic curves were used to determine *C*_min_ cut‐offs for AEs.

**Results:**

A total of 401 patients were included. Among 108 patients with high initial *C*_min_, voriconazole was discontinued in 32 and the dose was reduced in 71. Among 44 patients with low initial *C*_min_, voriconazole was discontinued in 4 and the dose was increased in 19. Hepatotoxicity occurred in 6.0% of patients, after a median of 10 days. Visual symptoms were evident in 9.5% of patients after a median of 4 days. Initial *C*_min_ was significantly associated with visual symptoms but not hepatotoxicity, which suggested the effect of treatment modification on hepatotoxicity. However, both hepatotoxicity and visual symptoms were significantly correlated with *C*_min_ at the onset of AEs, and the *C*_min_ cut‐offs were 3.5 μg/mL for hepatotoxicity and 4.2 μg/mL for visual symptoms. Voriconazole was discontinued after the occurrence of AEs in 62.5% of patients with hepatotoxicity but only 26.3% of patients with visual symptoms. With dose adjustment, treatment was completed in 8/9 patients with hepatotoxicity and 27/28 patients with visual symptoms.

**Conclusions:**

A significant preventive effect was demonstrated on hepatotoxicity, but not on visual symptoms because of earlier occurrence. With treatment modification after the occurrence of AEs, most patients completed therapy.

## 1 INTRODUCTION

Voriconazole is an antifungal agent that is used to treat invasive aspergillosis and invasive candidiasis.^1^, ^2^ It is associated with adverse side effects including visual symptoms, neurological disorders and hepatotoxicity.^3^, ^4^, ^5^, ^6^, ^7^, ^8^ Xing et al^9^ comprehensively compared voriconazole‐induced toxicity with other antifungals, and there were significant differences in hepatotoxicity (odds ratio [OR] 1.60), visual symptoms (OR 6.50) and neurotoxicity (OR 1.99). Therapeutic drug monitoring (TDM) is used to guide voriconazole therapy to prevent drug‐related adverse events and to improve clinical responses by individualising dose regimens.^10^, ^11^

Park et al^12^ recently demonstrated the clinical efficacy of TDM in patients taking voriconazole in a randomised clinical study. Voriconazole TDM significantly reduced drug discontinuation due to adverse effects, and a higher proportion of patients achieved a clinical response with TDM compared to the non‐TDM group. This is particularly important for Asian populations. Voriconazole is metabolised primarily via CYP2C19, and to a lesser extent CYP3A4 and CYP2C9.^13^ Allelic polymorphism of CYP2C19 has been reported, and CYP2C19 non–wild‐type alleles are generally found in 60%‐70% of people in Asian populations but only 30% of Caucasian and African Americans.^14^ Voriconazole concentrations can be as much as four times higher in individuals with non–wild‐type alleles than in those with wild‐type alleles.^15^, ^16^ These observations imply that Japanese individuals taking voriconazole are at a comparatively high risk of hepatotoxicity because of their genetic background. Jin et al^17^ conducted a systematic review and meta‐analysis to determine the optimal voriconazole trough concentration (*C*_min_) and reported that *C*_min_ > 3 μg/mL was associated with increased hepatotoxicity in Asians but not in other races.

In many institutions, monitoring serum voriconazole concentrations has become routine, and antimicrobial stewardship programmes incorporate TDM. Notably however, few studies have assessed the effects of antifungal stewardship using voriconazole TDM in real clinical settings. The aim of the current study was to evaluate the influence of treatment modification of voriconazole based on initial *C*_min_ on the prevention of adverse effects, and the capacity of treatment modification after the occurrence of adverse effects to enable continued effective voriconazole therapy in Japanese patients.

## 2 PATIENTS AND METHODS

### 2.1 Setting

Patients aged ≥18 years who were receiving voriconazole were being monitored by an antimicrobial stewardship team and had had their voriconazole *C*_min_ measured at least once during therapy was eligible for inclusion. Exclusion criteria were prophylactic use of voriconazole and unconsciousness precluding the determination of visual symptoms. This retrospective study was conducted at five hospitals in Japan between April 2015 and March 2018. Medical records were individually reviewed at each study site using a standardised data collection template to collect demographic information and clinical data on adverse events, as well as voriconazole dosing information. Hepatotoxicity was evaluated using laboratory data obtained at least once a week or at the time of TDM. Proven invasive fungal infection and probable/possible fungal infection were diagnosed in accordance with previously reported criteria.^18^, ^19^, ^20^

### 2.2 Dosage adjustment and therapeutic drug monitoring

An adequate voriconazole dose was defined as a loading dose of 5‐6 mg/kg twice daily followed by a maintenance dose of 3‐4 mg/kg twice daily. The maintenance dose was decreased to 1.5‐2 mg/kg in patients with liver dysfunction (Child‐Pugh A‐C).^21^ Because the dose was calculated on the basis of body weight, dose rounding within 10% of the recommended dose was considered as appropriate. Optimal timing of TDM was defined as 4‐10 days after the start of therapy.^21^ Voriconazole concentrations were measured using high‐performance liquid chromatography. The lower limit of quantification of the assay was 0.1 μg/mL; therefore, <0.1 μg/mL was recorded as zero for the purposes of data analysis. The target voriconazole *C*_min_ was set at 1‐5 µg/mL in the current study (≥1‐2 µg/mL for efficacy and <4‐5 µg/mL to prevent adverse effects^21^). Relationships between well‐known concentration‐dependent adverse effects including hepatic dysfunction and visual symptoms and *C*_min_ (initial *C*_min_ and *C*_min_ at the onset of adverse effects) were analysed.

### 2.3 Adverse effects

Elevations in liver function test results including alanine aminotransferase (ALT), aspartate aminotransferase (AST), alkaline phosphatase, gamma‐glutamyl transpeptidase and bilirubin were recorded, and adverse events were graded in accordance with the *Common Terminology Criteria for Adverse Events version 5.0*.^22^ Hepatic dysfunction was defined as AST or ALT levels at or above three times the upper limit of normal. If the AST or ALT baseline was abnormal, hepatic dysfunction was defined as AST or ALT at or above three times the baseline. Any visual symptoms during therapy including changes in colour perception, blurred vision, bright spots, wavy lines and photophobia were considered to be adverse effects caused by voriconazole. Visual hallucinations are usually classified as a symptom of neurotoxicity, but because clearly differentiating them from visual symptoms can be challenging, they were included as visual symptoms in the current study.

### 2.4 Statistical methods

The data were expressed as medians and interquartile ranges (IQRs). The chi‐squared test was used to analyse categorical data, and the paired *t* test was used to analyse continuous data. Statistical analysis was performed with spss ver. 24 (SPSS Inc, Chicago, IL, USA). *P* < .05 was deemed to indicate statistical significance. Cut‐off values were the maximum area under the curve (AUC) as determined via a receiver operating characteristic (ROC) curve.

## 3 RESULTS

### 3.1 Patient characteristics

Overall, data from 583 patients were reported. Of these, 182 in whom voriconazole use was prophylactic were excluded from the study, and 401 in whom voriconazole was used for treatment were included. The median duration (IQR) of follow‐up was 48 days (23‐129). Patients' demographic and clinical characteristics are shown in Table 1. There were 99/401 (24.7%) patients with proven invasive fungal infection (candidiasis 46, aspergillosis 37, cryptococcosis 9 and ‘other’ 8. One patient had two different fungal infections. There were a further 209 (52.1%) patients with probable/possible fungal infections, and in 93 (23.2%) patients, no fungal infections were diagnosed. Haematological malignancy was the most common underlying condition (39.2%). The routes of initial administration were intravenous in 119 patients and oral in 282 patients. Intravenous administration was selected in 111/340 patients (32.6%) with estimated glomerular filtration rates ≥30 and 2/26 patients (7.7%) with estimated glomerular filtration rates <30, and in 6/35 patients (17.1%) who underwent intermittent haemodialysis or continuous renal replacement therapy. Oral step down was performed in 48/119 patients (40.3%) in whom intravenous administration was selected initially. The median duration (IQR) of voriconazole treatment was 30 days (14‐117).

**TABLE 1:** Patient demographic and clinical characteristics

Factors	Total population of the study (n = 401)
Age (range)	61.8 ± 15.5 (18‐91)
Male/female, no. of patients	228/173
Body weight (range)	51.7 ± 10.5 (28.6‐110.6)
Primary disease, no. of patients (rate)	
Haematological malignancy	157 (39.2%)
Collagen disease	88 (21.9%)
Solid organ malignancy	39 (9.7%)
Benign respiratory tract disease	23 (5.7%)
Diabetes	17 (4.2%)
Inflammatory bowel disease	17 (4.2%)
Skin and soft tissue disease	12 (3.0%)
Solid organ transplantation	4 (1.0%)
Liver cirrhosis	13 (3.2%) (Child‐Pugh A, 2; B, 4; C, 7)
Chronic renal disease	7 (1.7%)
Neurological disease	6 (1.5%)
Other	31 (7.7%)
Diagnosis of the fungal disease, no. of patients (rate)	
Proven	99 (24.7%)
Candidiasis	46
Aspergillosis	37 (coinfection with cryptococcosis: 1)
Cryptococcosis	9
Other	8
Probable/possible	209 (52.1%)
Undiagnosed (empirical therapy)	93 (23.2%)
Route of administration in initial therapy	
Intravenous	119 (29.7%)
Oral	282 (70.3%)

### 3.2 Dosage adjustment and timing of initial therapeutic drug monitoring

Loading doses were administered to 65.8% of patients. The median dose (IQR) on the initial day of treatment was 5.9 mg/kg twice daily (5.4‐6.1) in patients who received a loading dose. The median maintenance dose was 3.8 mg/kg twice daily (IQR 3.2‐4.1) and the rate of adherence to the standard dose was 75.1%. Low adherence to a reduced dose (3/13 patients, 23.1%) was observed in patients with liver cirrhosis. The median day of TDM after the start of therapy was 6 (IQR 5‐7), and the rate of adherence to adequate timing was 88.5%. TDM was performed a median (IQR) of two times (1‐4) for each patient. The rate of adherence to both the recommended dosing regimen and the recommended TDM timing was 56.4% (226/401 patients).

### 3.3 Voriconazole trough concentration

The median initial *C*_min_ was 3.33 μg/mL (IQR 1.90‐5.13). In patients with adequate dosing including the loading dose and adequate TDM timing, the median initial *C*_min_ (IQR) was 3.91 μg/mL (2.50‐5.48). In total, 29.6% of patients had high *C*_min_ (≥5 μg/mL), whereas only 6.6% of patients had low *C*_min_ (<1 μg/mL, Table 2). In 26 patients with sequential therapy (intravenous to oral administration and oral to intravenous administration) whose dose was not altered when the administration route was changed, *C*_min_ was significantly lower in patients who were treated orally than in those treated intravenously (median [IQR]: 2.30 [1.50‐2.76] µg/mL vs 3.00 [2.19‐3.40] µg/mL, *P* = .005), and the median oral bioavailability (IQR) was 83.9% (74.2‐90.4). In an individual patient‐based comparison, *C*_min_ was lower in all patients treated orally, excluding one patient who developed herpes zoster after switching oral therapy. This patient was treated with acyclovir, acetaminophen and pregabalin, and worsening of liver function, which might influence the voriconazole concentration was not observed (Figure S1). The median *C*_min_ was 4.26 µg/mL in patients with Child‐Pugh A and B, vs 3.57 µg/mL in patients with Child‐Pugh C. In patients without maintenance dose reduction, median *C*_min_ was 4.26 and 3.79 µg/mL, respectively.

**TABLE 2:** Initial voriconazole trough concentration (*C*_min_)

Initial *C*_min_	Total (n = 401)	Adequate dosing and timing of TDM (n = 226)
Median (interquartile range) (μg/mL)	3.33 (1.90‐5.13)	3.91 (2.50‐5.48)
*C*_min_ categories		
<1 μg/mL	44 (11.0%)	15 (6.6%)
1‐5 μg/mL (target concentration range)	249 (62.1%)	144 (63.7%)
≥5 μg/mL	108 (26.9%)	67 (29.6%)

Abbreviation: TDM, therapeutic drug monitoring.

### 3.4 Modification for voriconazole treatment according to initial trough concentration

Some modification was performed in 103/108 patients (95.4%) with high initial *C*_min_ (discontinuation 32, dose reduction 71) and in 23/44 patients (52.3%) with low initial *C*_min_ (discontinuation 4, dose increase 19). Subsequent TDM was performed in 89.5% of patients who underwent dose reductions, all patients who underwent dose increases, and 70.5% of patients who did not undergo any dose adjustment. With regard to the effects of dose adjustment, *C*_min_ subsequently reached the target range (1‐5 μg/mL) in 87.0% of patients with dose reductions, and 88.0% of patients with dose increases. In total, 87.3% of patients who underwent dose adjustment subsequently achieved a *C*_min_ within the target range (Table 3).

**TABLE 3:** Subsequent trough concentration (*C*_min_) according to voriconazole dose adjustment based on initial *C*_min_

Dose adjustment	Subsequent *C*_min_ (μg/mL)				
	<1	1‐5	≥5	Total	No data available
Dose adjustment (n = 120)	3 (2.7%)	96 (87.3%)	11 (10.0%)	110 (100%)	10
Dose reduction (n = 95)	1 (1.2%)	74 (87.0%)	10 (11.8%)	85 (100%)	10
Dose increase (n = 25)	2 (8.0%)	22 (88.0%)	1 (4.0%)	25 (100%)	0
Same dose (n = 220)	18 (11.6%)	128 (82.6%)	9 (5.8%)	155 (100%)	65

### 3.5 Adverse effects and voriconazole trough concentration

Hepatotoxicity occurred in 24 patients (6.0%), and visual symptoms were reported by 38 patients (9.5%) (photophobia 14, visual hallucination 7, altered colour perception 6, blurred vision 5, visual field abnormality 4, bright spots 3, wavy lines 3). An opioid that can also result in visual symptoms was administered concomitantly in one patient who complained of visual symptoms. The rates of voriconazole discontinuation because of a drug‐related adverse effect were 2.5% (10/401) in the hepatotoxicity group and 2.7% (11/401) in the visual symptom group. The median timepoints of adverse effect onset after the start of therapy were day 10 for hepatotoxicity and day 4 for visual symptoms (*P* < .001). Adverse effects occurred 8 days after voriconazole initiation or earlier in 50.0% of patients with hepatotoxicity and 81.6% of patients with visual symptoms (*P* = .009). In the total study population, the incidence of early occurrence (≤8 days) of adverse effects in patients with visual symptoms was significantly higher than that in patients with hepatotoxicity (7.7% vs 3.0%, *P* = .003).

**FIGURE 1:**
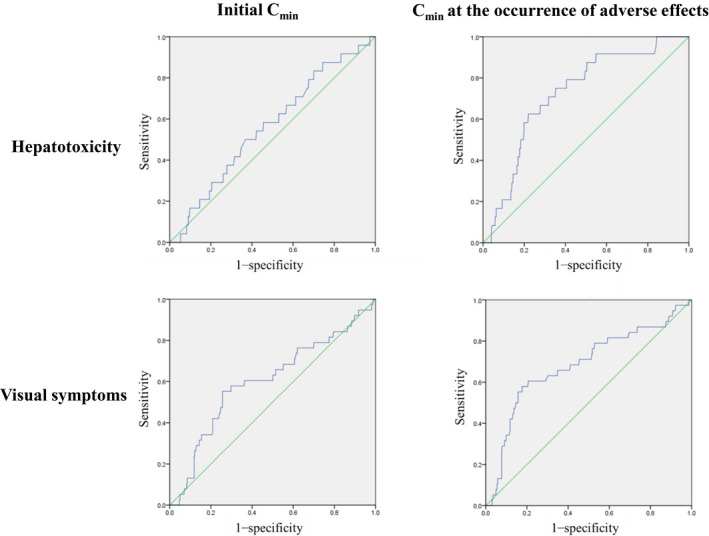
Receiver operating characteristic curve of initial trough concentration (*C*_min_) and *C*_min_ at the occurrence of adverse effects to predict adverse effects

The ROC curve of initial *C*_min_ and *C*_min_ at the onset of adverse effects used to predict adverse effects (*C*_min_ at last TDM during therapy was used in patients without adverse effects) is shown in Figure 1. Although higher initial *C*_min_ was associated with visual symptoms (AUC 0.603, cut‐off 4.9 μg/mL, OR 3.59, *P* = .037), there was no significant correlation between hepatotoxicity and initial *C*_min_ (AUC 0.562, cut‐off 3.6 μg/mL, OR 1.67, *P* = .292). In contrast, *C*_min_ at the onset of adverse effects was significantly associated with hepatotoxicity (AUC 0.725, OR 5.20, *P* < .001) and visual symptoms (AUC 0.684, OR 5.89, *P* < .001), and the *C*_min_ cut‐offs for predicting the occurrence of adverse effects were 3.5 μg/mL for hepatotoxicity and 4.2 μg/mL for visual symptoms.

Voriconazole was discontinued within 3 days in 15/24 patients (62.5%) with hepatotoxicity and 10/38 patients (26.3%) with visual symptoms (*P* = .005). Among patients in whom voriconazole was continued, the dose was reduced in 8/9 patients (88.9%) with hepatotoxicity. In contrast, dose reduction was only performed in 11/28 patients (39.3%) who reported visual symptoms (*P* = .019).

In patients with ≥5 μg/mL of *C*_min_ at the onset of adverse effects, dosages were reduced in 62.5% of patients with hepatotoxicity and 57.9% of patients who reported visual symptoms. Voriconazole was discontinued in 75.0% of patients with hepatotoxicity who had a *C*_min_ 1‐5 μg/mL, because there was no subsequent target *C*_min_ for dose adjustment. In contrast, voriconazole was only discontinued in 23.5% of patients who reported visual symptoms and had a *C*_min_ 1‐5 μg/mL. After dose reduction, all patients with hepatotoxicity and 90.0% of patients who reported visual symptoms subsequently achieved a *C*_min_ within the target range. Adverse effects were improved, and voriconazole therapy was completed in 8/9 patients with hepatotoxicity (88.9%) and 27/28 patients who reported visual symptoms (96.4%). One patient in whom visual symptoms did not improve had concomitantly used opioids. In seven patients with visual hallucination, the initial median *C*_min_ was 4.87 μg/mL. Although voriconazole was continued (the same dose in six patients and dose reduction in one patient), visual hallucination improved in all patients.

## 4 DISCUSSION

Achieving the target *C*_min_ is important for preventing adverse effects and improving clinical efficacy.^21^ Pascual et al^23^ reported that median *C*_min_ was 2.9 μg/mL in patients on ≥8 mg of voriconazole per day, and 1.7 μg/mL in patients on 7 mg/kg per day. Racil et al^24^ reported that half of the patients had a median *C*_min_ of <1.0 μg/mL, and *C*_min_ was only >5.0 μg/mL in 3.1% of patients. Compared with these previous reports, higher *C*_min_ was evident in Japanese patients who fulfilled standard dosing criteria and those with TDM obtained at an appropriate time in the current study, a third of patients had high *C*_min_, and only 5% had low *C*_min_. These results suggest that TDM should be mandatory in Japanese patients who are prone to adverse effects when they exhibit high voriconazole concentrations.

In the present study, there was a clear correlation between adverse effects and *C*_min_ at the onset of adverse effects, and the *C*_min_ cut‐offs for predicting adverse effects were 3.5 μg/mL for hepatotoxicity and 4.2 μg/mL for visual symptoms. Notably however, there was no significant association between initial *C*_min_ and the subsequent development of hepatotoxicity. Chu et al^25^ reported that there was no association between increased hepatotoxicity at voriconazole levels >5.5 mg/L. In another multicentre study that included 264 patients with haematological diseases, there was also no correlation between *C*_min_ and voriconazole toxicity.^24^ Those studies raise the question of the utility of routine clinical monitoring of voriconazole plasma concentrations. Notably however, those results and the results of the present study should be assessed with caution. Treatment modification including dose adjustment based on initial *C*_min_ may prevent adverse effects that could subsequently occur thereafter. In the current study, treatment modification was conducted in almost all patients with high initial *C*_min_. After dose adjustment, *C*_min_ within the target range was achieved in approximately 90% of patients. As a consequence, only 6.0% of patients in the present study had hepatotoxicity, which is much lower than previously reported frequencies.^3^, ^4^, ^26^, ^27^ In a multicentre study conducted by Saito et al,^3^ 16.9% of treated patients had hepatotoxicity. Luong et al^4^ reported that 51% of patients who were treated with voriconazole developed hepatotoxicity, and 34% of them had to discontinue the treatment for that reason. In a systematic review and meta‐analysis, Xing et al^9^ reported that hepatotoxicity occurred in 17.7% of patients who underwent voriconazole therapy.

In the present study, there was a significant correlation between initial *C*_min_ and visual symptoms, possibly because of the comparatively earlier occurrence of this adverse effect. TDM was conducted a median of 6 days after the initiation of voriconazole treatment, *C*_min_ was usually recorded 2 days later, and in total, at least 8 days was required before the results of TDM were observed. Given the comparatively early onset of visual symptoms, treatment modification arising from initial TDM results before the occurrence of adverse effects could not be conducted in approximately 80% of patients who reported visual symptoms. Other authors have also reported that visual symptoms and hallucinations tended to occur during the first week of therapy and that symptoms were reduced or disappeared despite continued therapy in most patients.^8^, ^28^, ^29^

As antifungal stewardship for patients with adverse effects, voriconazole discontinuation or dose reduction was more frequently performed in patients with hepatotoxicity than in those who reported visual symptoms, and natural remission of visual symptoms was expected. With dose reduction, all patients with hepatotoxicity subsequently achieved a *C*_min_ that was within the target range, and hepatotoxicity was improved in 88.9% of patients. In contrast, visual symptoms were improved irrespective of dose adjustment in almost all patients.

The present study had some limitations. First, this was a retrospective study in a defined population, and the results may not be applicable to other populations. Second, polymorphisms in CYP2C19 that are known to influence drug metabolism^14^ were not determined in the study. Third, patients with candidiasis, aspergillosis and cryptococcosis and those with empirical therapy were all included in the study, rendering the study sample relatively heterogeneous in this regard. The outcomes according to the voriconazole plasma concentration may differ among patients with these types of fungal infections,^30^ and relationships between *C*_min_ and clinical success were not evaluated. Finally, although no previous studies evaluated the safety of voriconazole in patients with Child‐Pugh C severe liver disease, we included seven patients with Child‐Pugh C disease.

In conclusion, in the current study the high performance of voriconazole treatment modification based on initial *C*_min_ by way of an antifungal stewardship programme was confirmed in clinical practice in Japan, and the results of the study suggest that it may promote a low incidence of hepatotoxicity. The chance of optimising voriconazole levels based on the initial TDM result was low in patients who reported visual symptoms, however, because of their comparatively earlier onset. In addition to the above‐described preventive effects on hepatotoxicity, modification of therapy after the occurrence of adverse effects had a substantial capacity to facilitate continuation and completion of voriconazole therapy.

## CONFLICT OF INTEREST

Y. Takesue has received grant support from Sumitomo Dainippon Pharma Co., Ltd., and Shionogi & Co., Ltd., and payment for lectures from Astellas Pharma Inc, and MSD Japan. T. Miyazaki has received grants from Pfizer and MSD and lectures honoraria from Pfizer, MSD, Sumitomo Dainippon Pharma and Astellas Pharma. Y. Miyazaki has received payment for lectures from Astellas Pharma Inc, Sumitomo Dainippon Pharma Co. Ltd. and MSD Japan. Other authors have no conflict of interest to declare.

## AUTHOR CONTRIBUTIONS

**Yukihiro Hamada:** Investigation (equal); Writing‐original draft (supporting). **Takashi Ueda:** Formal analysis (lead); Investigation (equal); Methodology (lead); Writing‐original draft (equal); Writing‐review & editing (equal). **Yoshitsugu Miyazaki:** Supervision (equal). **Kazuhiko Nakajima:** Investigation (supporting). **Keiko Fukunaga:** Investigation (equal). **Taiga Miyazaki:** Investigation (supporting). **Nana Nakada‐Motokawa:** Investigation (equal). **Miki Nagao:** Investigation (equal). **Hideki Kawamura:** Investigation (supporting). **Akari Shigemi:** Investigation (equal). **Fumiya Ebihara:** Investigation (equal). **Toshimi Kimura:** Investigation (supporting). **Kazuhiro Ikegame:** Investigation (supporting). **Motoi Uchino:** Investigation (supporting). **Hiroki Ikeuchi:** Investigation (supporting). **Yoshio Takesue:** Conceptualization (equal); Project administration (equal); Writing‐original draft (lead); Writing‐review & editing (lead).

## ETHICAL APPROVAL

The study was approved by the institutional review boards of Hyogo College of Medicine (No. 2996), Tokyo Women's Medical University Hospital (No. 5012), Nagasaki University Hospital (No. 18111912), Kyoto University Hospital (No. E2300) and Kagoshima University (No. 180225).

## Supporting information

Fig S1
