# Supplementary material for: Effects of antifungal stewardship using therapeutic drug monitoring in voriconazole therapy on the prevention and control of hepatotoxicity and visual symptoms: A multicentre study conducted in Japan
Source: Mycoses. 2020 Jun 25;63(8):779–86. doi: 10.1111/myc.13129 (PMC7496238; doi:10.1111/myc.13129)
Supplement: Supplementary file 1 — Fig S1 [file MYC-63-779-s001.pptx]

## Slide 1
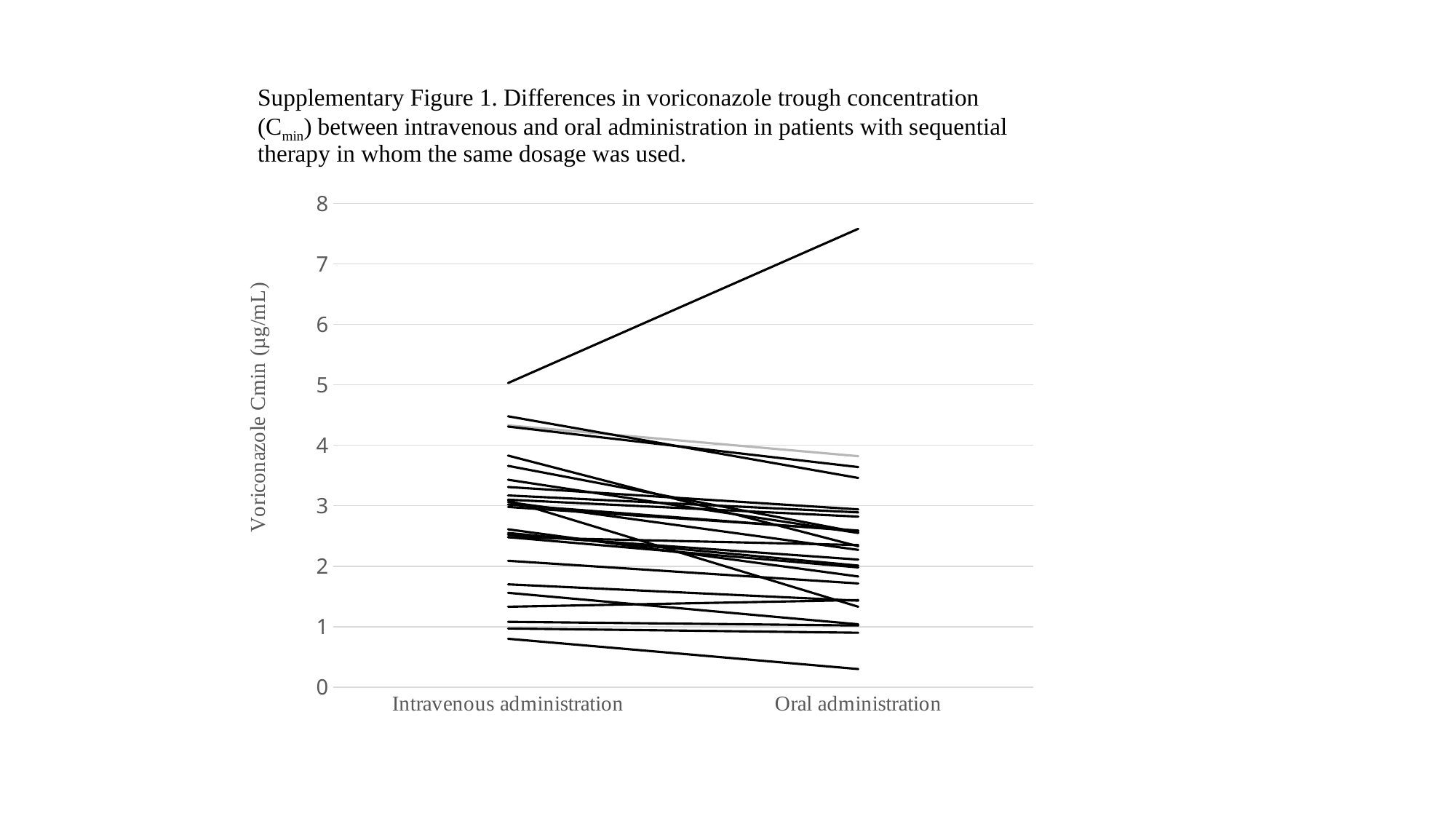

# Supplementary Figure 1. Differences in voriconazole trough concentration (Cmin) between intravenous and oral administration in patients with sequential therapy in whom the same dosage was used.
### Chart
| Category | | | | | | | | | | | | | | | | | | | | | | | | | | |
|---|---|---|---|---|---|---|---|---|---|---|---|---|---|---|---|---|---|---|---|---|---|---|---|---|---|---|
| Intravenous administration | 2.09 | 3.02 | 3.17 | 3.09 | 2.55 | 3.43 | 3.8299999999999996 | 3.31 | 2.52 | 3.1 | 3.06 | 2.61 | 2.48 | 3.66 | 4.33 | 1.56 | 2.48 | 1.08 | 1.33 | 1.7 | 4.48 | 0.97 | 0.8 | 5.03 | 4.31 | 2.98 |
| Oral administration | 1.715 | 2.58 | 2.89 | 1.33 | 2.01 | 2.55 | 2.33 | 2.94 | 2.11 | 2.82 | 2.27 | 1.83 | 1.98 | 2.56 | 3.82 | 1.04 | 2.35 | 1.02 | 1.44 | 1.43 | 3.46 | 0.9 | 0.3 | 7.58 | 3.64 | 2.59 |
